# Initial Recurrence Risk Stratification of Papillary Thyroid Cancer based on Intratumoral and Peritumoral Dual Energy CT Radiomics

**DOI:** 10.2174/0115734056402179250813050300

**Published:** 2025-08-21

**Authors:** Yan Zhou, Yongkang Xu, Yan Si, Feiyun Wu, Xiaoquan Xu

**Affiliations:** 1 Department of Radiology, The First Affiliated Hospital with Nanjing Medical University, Nanjing, China; 2 Department of Thyroid Surgery, The First Affiliated Hospital with Nanjing Medical University, Nanjing, China

**Keywords:** Papillary thyroid cancer, Radiomics, Machine learning, Multidetector computed tomography, Prognosis, Dual-energy computed tomography, American thyroid association

## Abstract

**Introduction:**

This study aims to evaluate the potential of Dual-Energy Computed Tomography (DECT)-based radiomics in preoperative risk stratification for the prediction of initial recurrence in Papillary Thyroid Carcinoma (PTC).

**Methods:**

The retrospective analysis included 236 PTC cases (165 in the training cohort, 71 in the validation cohort) collected between July 2020 and June 2021. Tumor segmentation was carried out in both intratumoral and peritumoral areas (1 mm inner and outer to the tumor boundary). Three region-specific rad-scores were developed (rad-score [VOI^whole^], rad-score [VOI^outer layer^], and rad-score [VOI^inner layer^]), respectively. Three radiomics models incorporating these rad-scores and additional risk factors were compared to a clinical model alone. The optimal radiomics model was presented as a nomogram.

**Results:**

Rad-scores from peritumoral regions (VOI^outer layer^ and VOI^inner layer^) outperformed the intratumoral rad-score (VOI^whole^). All radiomics models surpassed the clinical model, with peritumoral-based models (radiomics models 2 and 3) outperforming the intratumoral-based model (radiomics model 1). The top-performing nomogram, which included tumor size, tumor site, and rad-score (VOI^inner layer^), achieved an Area Under the Curve (AUC) of 0.877 in the training cohort and 0.876 in the validation cohort. The nomogram demonstrated good calibration, clinical utility, and stability.

**Discussion:**

DECT-based intratumoral and peritumoral radiomics advance PTC initial recurrence risk prediction, providing clinical radiology with precise predictive tools. Further work is needed to refine the model and enhance its clinical application.

**Conclusion:**

Radiomics analysis of DECT, particularly in peritumoral regions, offers valuable predictive information for assessing the risk of initial recurrence in PTC.

## 1 INTRODUCTION

Papillary Thyroid Carcinoma (PTC) is one of the most common types of thyroid cancer. Accurate assessment of initial recurrence risk in PTC is the cornerstone of individualized clinical management [1, 2]. This risk helps guide decisions on the scope of thyroid surgery, the necessity and scope of neck lymph node removal, and the follow-up schedule [1]. The current initial recurrence risk stratification system recommended by the American Thyroid Association (ATA) guidelines mainly depends on pathological characteristics [1]. However, these characteristics can only be obtained postoperatively, lagging behind the surgical plan making. Therefore, accurate preoperative prediction of initial recurrence risk is of deep clinical need.

Several previous studies have tried to use clinical risk factors and tumor radiographic features to predict recurrence risk in PTC [3-8]. They found that patient age, history of Hashimoto thyroiditis, and tumor microcalcifications correlated positively with recurrence [3-8]. However, these studies concluded with inconsistent results, and these radiographic features were subjective. Moreover, they ignored tumor heterogeneity, which is closely associated with the biological behavior of the target tumor [7, 8].

Radiomics is a powerful technique for exploring tumor heterogeneity through extracting and quantifying large amounts of features from medical images, aiding in tumor differential diagnosis, prognosis prediction, and therapy evaluation [9-11]. Compared to ultrasound, contrast-enhanced Computed Tomography (CT) offers superior capability for evaluating retropharyngeal lymph node involvement and delineating primary tumor relationships with surrounding anatomical structures. CT combined with ultrasound is recommended for accurate preoperative evaluation in PTC [1, 12]. Previous studies have confirmed that radiomics analysis of conventional CT was effective in diagnosing or predicting some invasive pathological characteristics in PTC [13-15].

Unlike conventional CT, Dual-Energy CT (DECT) provides spectral tissue characterization and generates different image types through additional image reconstructions [16-18]. It can decompose materials and generate iodine maps, which directly quantify content and figure the distribution of the tissue contrast media [18]. Since iodine maps are closely correlated with tumor perfusion, they may serve as an imaging biomarker for tumor vascularity [18-20]. Previous studies have demonstrated that DECT-based radiomics can assist in predicting lymph node metastasis and extrathyroidal extension in PTC, with better predictive power compared to conventional CT [19, 20].

Although DECT-based radiomics has shown promise in exploring PTC heterogeneity, some limitations remain [19, 20]. First, prior studies have focused on a single invasive pathological characteristic, such as lymph node metastasis or extrathyroidal extension. Initial recurrence risk assessment in PTC is more comprehensive, encompassing not only these pathological characteristics but also factors such as the response to therapy (*e.g*., thyroglobulin levels). Second, these studies have primarily performed radiomics analysis only in the whole tumor region, whereas ignoring the peritumoral information. Previous studies have proven that tumor peritumoral image features are more favorable for evaluating tumor biological behavior, warranting further investigation [21-24].

Therefore, this study aimed to conduct DECT-based radiomics in both intratumoral and peritumoral regions to preoperatively predict the initial recurrence risk in PTC.

## 2 MATERIALS AND METHODS

### 2.1 Study Population

**Fig. (1):**
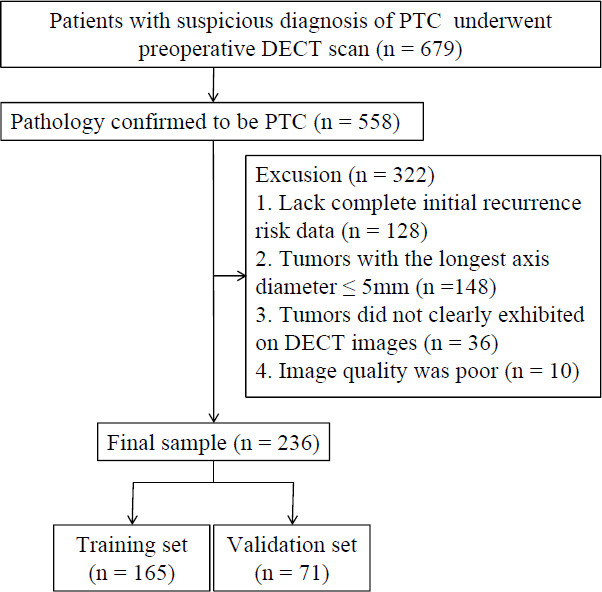
Patient flowchart.

This retrospective study received approval from our institutional review board, which waived the requirement for informed consent. Medical records were collected for 679 consecutive patients suspected of having PTC who had a preoperative DECT scan between July 2020 and June 2021. Patients were included based on these criteria: (1) histopathologically confirmed PTC diagnosis following surgical resection, (2) availability of complete initial recurrence risk data for evaluation, (3) tumors with the longest axis diameter > 5mm to ensure sufficient volume for analysis, (4) tumors clearly exhibited on DECT images, and (5) adequate DECT images quality for subsequent analysis. Patients were excluded if they met any of the following criteria: (1) patients with a pathology diagnosis other than PTC (n = 121), (2) lack of complete initial recurrence risk data (n = 128), (3) tumors with the longest axis diameter ≤ 5mm (n = 148), (4) tumors that were not clearly exhibited on DECT images (n = 36), and (5) poor image quality (n = 10). After applying these inclusion and exclusion criteria, the final study cohort consisted of 236 patients (Fig. **1**). The primary study cohort was allocated into a training (165 patients) and a validation set (71 patients) based on the time of surgery.

### 2.2 Initial Recurrence Risk Stratification

Patients were stratified into low, intermediate, and high risk of initial recurrence based on the initial recurrence risk stratification system established in the 2015 ATA guidelines [1]. Low-risk patients referred to these who had no radiographic or pathologic evidence of extrathyroidal extension, vascular invasion, or distant metastases, with lymph nodes involvement of < 5 lymph nodes containing micro metastases (< 0.2cm). Intermediate-risk patients had either aggressive histology, minimal extrathyroidal extension, vascular invasion, detectable iodine uptake outside the thyroid bed, or > 5 involved lymph nodes (0.2-3cm). High-risk patients demonstrated gross extrathyroidal extension, incomplete tumor resection, distant metastases, or involved lymph nodes (> 3cm).

According to the methods, patients were classified as low risk (n = 128), intermediate risk (n = 76), or high risk (n = 32). For low-risk patients, a thyroid lobectomy is considered sufficient, while those with intermediate or high recurrence risk require a total thyroidectomy and closer follow-up. Therefore, intermediate and high-risk patients were grouped together for binary classification [1]. Finally, patients were split into: (1) low-risk patients (training set, n = 86; validation set, n = 42) and (2) intermediate/high-risk patients (training set, n = 79; validation set, n = 29).

### 2.3 DECT Imaging Acquisition and Post-Processing

The DECT scanning parameters and computational analysis methods are summarized in Supplementary Methods S1. Six different image sets were ultimately reconstructed for subsequent analysis, including unenhanced, arterial, and venous phase mixed images, as well as iodine maps.

### 2.4 Clinical Data

Demographic and clinical characteristics extracted from medical records comprised age, sex, Body Mass Index (BMI), presence of nodular goiter, and Hashimoto thyroiditis. Age stratification employed cut-off values of 45 and 55 years. BMI was derived using the standard formula: weight (kg) / (height × height) (m^2^).

### 2.5 Tumor Conventional CT Image Features

Radiologists 1 and 2 assessed tumor conventional CT image features on mixed images [19, 20]. If a disagreement occurred, Senior Radiologist 3 would evaluate and decide the permanent results. All radiologists were blinded to the clinical design. If more than one lesion exists, only the largest one was analyzed. The radiologists assessed the following tumor morphological features: (1) size (axial): the longest diameter in the axial slice with the maximum tumor size, (2) size (coronal): the longest diameter in the coronal slice with the maximum tumor size, (3) location: categorized as right lobe, left lobe, and isthmus, (4) site (Position, S-I): categorized as superior, medium, and inferior, (5) site (Position, V-D): categorized as ventral, medium, and dorsal, (6) aspect ratio: the ratio of the long diameter to the short diameter in the axial slice showing the maximum tumor size, subclassified as one, (7) shape: round and quasi round were defined as regular, other shape was considered as irregular, (8) calcification: categorized as no calcification, macrocalcification, or microcalcification. Microcalcification was defined as calcification diameter ≤ 2mm, and (9) cystic [19, 20].

### 2.6 Intratumoral and Peritumoral Tumor Segmentation

The radiomics workflow of our study is depicted in Fig. (**2**). Radiologist 2 semi-automatically segmented both intratumoral and peritumoral tumor areas on mixed images and iodine maps using Syngo.via Frontier Radiomics software (Siemens Healthcare) [25]. The software used in this study provided automatic contouring capabilities combined with freehand drawing, powered by an integrated algorithm. This algorithm detected points along the tumor border and computed an ellipsoidal approximation of the tumor shape. By tracing the tumor contour, the entire volume of interest (VOI^whole^) was automatically generated [25, 26]. Fine adjustments to the VOI contours were made manually to exclude calcified and cystic regions. The software's object operations and combination module, which incorporates morphological operations such as erosion and dilation, enabled the automatic segmentation of the peritumoral regions at 1 mm distances both outside (VOI^outer layer^) and inside (VOI^inner layer^) the tumor boundary [24, 26]. This 1 mm margin was chosen to accommodate the small size of thyroid lesions on CT images while balancing segmentation accuracy with computational feasibility [19, 20]. All VOIs were reconfirmed by Radiologist 3. To evaluate intra-reader agreement, Radiologist 2 repeated the segmentation process at an interval of one month. A detailed process of intratumoral and peritumoral tumor segmentation is depicted in Fig. (**3**).

Radiomics features for each VOI were computed post-segmentation using the Syngo.via Frontier Radiomics software, integrated with the PyRadiomics library [27]. The feature extraction process encompassed 17 shape descriptors, 18 first-order statistical metrics, and 75 textural characteristics from the initial image data. To enhance feature detection, transformations such as squaring, square rooting, logarithmic, and exponential functions were applied, along with two Laplacian of Gaussian filters and eight wavelet decompositions. This comprehensive approach yielded a dataset of 1412 radiomics features per VOI for each image set.

### 2.7 Radiomics Features Selection and Rad-scores Building

We used an open-source software named Feature Explorer (FAE, version 0.2.7) for radiomics features selection and rad-scores building [28]. A 5-step procedure, including data normalization, consistency analysis, dimension reduction, feature selection, and classifier modeling, was devised to select radiomics features and build rad-scores. First, each radiomics data set was normalized to A unit with a 0 center. Second, interobserver feature reliability was assessed using the Intraclass Correlation Coefficient (ICC), with features demonstrating good reproducibility (ICC > 0.80) retained for further modeling. Third, Principal Component Analysis (PCA) was used for dimension reduction. Fourth, three feature selection methods, including Analysis of Variance (ANOVA), Recursive Feature Elimination (RFE), and relief, were used. Fifth, ten feature classifiers were utilized, including Linear Regression (LR), Support Vector Machine (SVM), Linear Discriminant Analysis (LDA), Auto-Encoder (AE), Random Forest (RF), Logistic Regression via Least Absolute Shrinkage and Selection Operator (LRLASSO), Ada-Boost (AB), Decision Tree (DT), Gaussian Process (GP), and Native Bayes (NB) [28].

To assess model robustness and stability, a 10-fold cross-validation was used, with stratification to preserve the distribution of the recurrence risk across each fold. The process was repeated 10 times, and the performance metrics were calculated for each repetition and averaged across all iterations [28]. Our machine learning pipeline, which systematically integrates these steps for feature selection, model building, and validation, is depicted in Fig. (**S1**) [29]. The optimal combination of feature selection method and classifier, which demonstrated the best predictive ability in both the training and validation sets, was chosen. Due to the three different types of VOIs, three radiomics scores (rad-score [VOI^whole^], rad-score [VOI^outer layer^], and rad-score [VOI^inner layer^]) achieving the optimal performance were finally selected.

### 2.8 Construction, Optimization, and Validation of Radiomics Models

Three different radiomics models (radiomics model 1-3) were constructed by incorporating the three radiomics scores and clinical risk predictors through multivariate logistic regression analyses, respectively [30, 31]. Additionally, a clinical model was built, comprising other pertinent risk factors except for the rad-scores. The predictive capabilities of all developed models were systematically assessed and compared. An independent validation set was used for internal validation.

### 2.9 A Radiomics Nomogram Establishment and Assessment

A radiomics nomogram was established based on the radiomics model that exhibited the best predictive performance, for providing an easy-to-use clinical tool. Model calibration was quantitatively evaluated through calibration curves with corresponding Hosmer-Lemeshow goodness-of-fit statistics [32]. To assess clinical usefulness, decision curve analysis was employed [33]. To verify the stability of the constructed nomogram, stratified analysis was performed in accordance with age, gender, and BMI.

### 2.10 Statistical Analysis

All statistical analyses were conducted using SPSS (v26.0, IBM) and MedCalc (v20.1). The data distribution was evaluated via Kolmogorov-Smirnov testing. Parametric continuous variables were expressed as mean ± Standard Deviation (SD) and compared using Student's t-test, while non-parametric data were presented as median (range) with Mann-Whitney U tests. Categorical variables were analyzed using χ^2^ or Fisher's exact tests, as appropriate. Model discrimination was evaluated through receiver operating characteristic (ROC) analysis (DeLong's method), with the Area Under the Curve (AUC) serving as the performance metric. A P value of less than 0.05 was regarded as significant.

## 3 RESULTS

### 3.1 Patient Characteristics

Detailed clinicopathological characteristics are summarized in Table **S1**. Baseline characteristics demonstrated comparable distributions between the training and validation cohorts (Tables **S2** and **S3**) (P > 0.05). Univariate analysis showed that tumor size (axial), tumor size (coronal), and tumor site (position, V-D) were significantly correlated with recurrence (Tables **1** and **2**).

### 3.2 Features, Mining, and Radiomics Scores Computation

A total of 3798 stable features with ICC higher than 0.80 were retained. The predictive performances of various machine learning algorithm combinations are summarized in Table **S4** and shown in Fig. (**S2A**-**F**). ANOVA combined with AE, ANOVA combined with LDA, and ANOVA combined with LDA had the optimal performance for VOI^whole^, VOI^outer layer^, and VOI^inner layer^, respectively. Ultimately, rad-score (VOI^whole^) containing 10 radiomics features, rad-score (VOI^outer layer^) containing 16 radiomics features, and rad-score (VOI^inner layer^) containing 17 radiomics features were constructed. The selected features and their corresponding weights in the rad-scores for VOI^whole^, VOI^outer layer^, and VOI^inner layer^ are shown in Fig. (**S3A**-**C**). The selected radiomics features from the three regions of interest provide valuable insights into tumor heterogeneity. Biological interpretations of the final selected individual radiomics features in the three rad-scores are provided in Table **S5**, and their performance is summarized in Table **S6**.

### 3.3 Intratumoral and Peritumoral Rad-scores Performance

Significant differences were observed in the three rad-scores between patients with low and intermediate/high recurrence risk (P all < 0.001) (Table **2**). Two presented cases using the three rad-scores for initial recurrence risk prediction are depicted in Fig. (**4**). The performance metrics were averaged over the 10-fold cross-validation procedure, which was repeated 10 times to ensure the model’s robustness. Peritumoral rad-scores (rad-score [VOI^outer layer^] and rad-score [VOI^inner layer^]) significantly outperformed the intratumoral rad-score [rad-score (VOI^whole^)] in both the training set (P = 0.036; P = 0.001) and the validation set (P = 0.044; P = 0.011). No significant differences were found between rad-score (VOI^outer layer^) and rad-score (VOI^inner layer^) (training set, P = 0.167; validation set, P = 0.820). The Rad-score (VOI^inner layer^) demonstrated the best predictive performance, achieving AUCs of 0.837 (95% CI: 0.772-0.890) in the training cohort and 0.843 (95% CI: 0.737-0.919) in the validation cohort. Detailed performances of the three rad-scores are summarized in Table **S7** and depicted in Fig. (**5A** and **B**).

### 3.4 Construction, Performance, and Validation of Different Models

A clinical model was constructed using tumor size (coronal) and tumor site (position, V-D). Three radiomics models were built, incorporating the constructed three rad-scores with tumor size (coronal) and tumor site (position, V-D). Key findings from the logistic regression modeling are tabulated in Table **3**.

All three radiomics-based models demonstrated statistically superior performance compared to the clinical model alone (training set, P = 0.018, P = 0.001, P < 0.001; validation set, P = 0.046, P = 0.025, P = 0.011). Notably, models incorporating peritumoral features (Models 2-3) significantly outperformed the intratumoral-only model (Model 1) in both sets (training, P = 0.039, P < 0.001; validation, P = 0.037, P = 0.034). The performance differences between radiomics model 2 and model 3 were not statistically significant (training set, P = 0.102; validation set, P = 0.762). Radiomics model 3, which combined tumor size (coronal), tumor site (position, V-D), and rad-score (VOI^inner layer^), achieved peak AUCs of 0.877 (training set) and 0.876 (validation set). Model comparisons are presented in Table **4** and visualized in Fig. (**5C** and **D**).

### 3.5 A Radiomics Nomogram Development and Assessment

A radiomics nomogram was established based on Radiomics Model 3, which includes tumor size (coronal), tumor site (position, V-D), and rad-score (VOI^inner layer^). This model demonstrated the highest predictive performance among the various models (Fig. **S4A**). Calibration of the established nomogram was favorable (Fig. **S4B**, **C**). Calibration testing confirmed the reliability of the nomogram (p > 0.05 in both sets). Clinically, the nomogram offered a significant net benefit over conventional approaches when treatment preference thresholds ranged from 0.165 to 0.682 risk probability (Fig. **S4D**, **SE**). Stratified analysis revealed that the performance of the radiomics nomogram remained favorable and stable across different subgroups, with AUCs consistently above 0.850. The results of the ROC curve analyses are summarized in Table **S8** and illustrated in Fig. (**S5A**-**H**).

## 4 DISCUSSION

In this study, we established three radiomics models based on intratumoral and peritumoral radiomics features derived from DECT to predict initial recurrence risk in PTC. All radiomics models outperformed the clinical model. Notably, the models incorporating peritumoral radiomics features overwhelmed the models based on intratumoral radiomics features. We further developed a radiomics nomogram incorporating tumor size, tumor site, and rad-score (VOI^inner layer^), which achieved the highest predictive performance for clinical practice. The nomogram's good calibration, clinical utility, and stable performance were also verified.

Clinicians are extremely concerned about the accurate evaluation of initial recurrence risk in PTC, which directly determines individualized treatment plans and follow-up intervals [1, 2]. Previous studies have indicated age, Hashimoto thyroiditis, and BMI as potential clinical risk factors for recurrence in PTC patients, though their findings have been inconsistent [3-6]. In our study, we found that larger tumor size (measured in coronal CT images) and tumor located in the medium position were pertinent risk factors for recurrence. Whereas these conventional features were subjective, and the clinical model based on them had limited predictive performance. Therefore, a more quantitative and accurate method is necessary for improving the predictive performance.

Radiomics can extract and quantify massive image features, aiding in tumor monitoring, prognosis prediction, and evaluation of the curative effect [9-11]. Previous studies have confirmed that radiomics can effectively predict invasive pathological characteristics in PTC patients [13-15]. However, they only paid attention to a single invasive pathological characteristic, limiting their ability to accurately assess prognosis. To compensate for this deficiency, our study constructed radiomics models combining DECT-based radiomics features with clinical risk factors to directly predict initial recurrence risk in PTC. We found that these radiomics models all exhibited favorable efficiency and performed significantly better than the clinical model. These results suggest that radiomics analysis, which reflects tumor heterogeneity, is a more objective and accurate method for predicting initial recurrence risk in PTC patients.

Given the critical role of the tumor microenvironment at the tumor periphery [21-24], we established three DECT-based rad-scores that incorporate both intratumoral and peritumoral regions extending 1 mm outward and inward from the tumor boundary. Notably, rad-score (VOI^outer layer^) and rad-score (VOI^inner layer^) both significantly outperformed rad-score (VOI^whole^). And the rad-score (VOI^inner layer^) achieved the highest performance. These findings suggest that radiomics analysis of the intratumoral region, reflecting the biological microenvironment at the tumor periphery, provides more valuable insights into recurrence risk prediction in PTC.

The majority of the radiomics features selected for the three rad-scores were derived from contrast-enhanced images. And over half of the selected radiomics features were derived from dual-phase iodine maps, which quantify and depict the distribution of iodine that is tightly associated with the heterogeneity of tumor perfusion and vascularity [18-20]. Biologically, iodine maps are particularly informative, as they reflect the tumor's blood supply, an essential factor in tumor progression. Tumors often exhibit abnormal blood vessel formation, resulting in irregular perfusion patterns. Radiomics features derived from dual-phase iodine maps capture the dynamic changes in iodine distribution, providing a more comprehensive understanding of the tumor's perfusion status and heterogeneity.

When compared to the study by Xu *et al*. [34], which used single-energy CT-based radiomics and reported AUCs of 0.746 to 0.754 for their combined models, our models showed superior performance. This can be attributed to two key factors: the use of DECT, which allows for better tissue differentiation and captures more detailed features of the tumor microenvironment, and the inclusion of both intratumoral and peritumoral regions. This dual approach offers a more comprehensive view of tumor heterogeneity, which is essential for accurate prediction of recurrence risk.

Our radiomics models can improve the current PTC diagnostic and treatment pipeline by providing a more accurate method for assessing recurrence risk. While clinicians traditionally rely on conventional imaging and clinical factors, these methods may not always accurately predict recurrence. By using DECT-based radiomics features from both intratumoral and peritumoral regions, our model offers a more comprehensive understanding of tumor heterogeneity across multiple scales. This could help guide more personalized treatment plans and follow-up schedules for PTC patients, improving clinical outcomes. Additionally, the nomogram we developed combines radiomics and clinical factors, making it a useful tool for everyday clinical practice.

## 5 LIMITATIONS

This study has several limitations. First, it was conducted with a relatively small sample size and at a single center, which may limit the generalizability of the findings. Multi-center studies with larger cohorts are needed to validate the results and improve the model's robustness. Second, the study did not incorporate ultrasound features, which are routinely used in clinical practice for the evaluation of PTC. Combining DECT radiomics with ultrasound features could further enhance the predictive performance. Third, although we employed real-time automatic tube current modulation (CARE dose 4D) to reduce radiation exposure during contrast-enhanced DECT, the potential risks associated with radiation exposure still warrant consideration. Future studies could explore low-dose DECT protocols or alternative imaging modalities to further minimize radiation exposure. Finally, the study lacked long-term follow-up data, which is crucial for assessing the accuracy of the models in predicting actual recurrence outcomes. A prospective design with extended follow-up would provide valuable insights into the clinical utility of our approach.

## CONCLUSION

In summary, intratumoral and peritumoral radiomics analysis of DECT is useful for predicting initial recurrence risk in PTC, especially when performed in the peritumoral region. The user-friendly radiomics nomogram, which integrates tumor morphological characteristics with peritumoral radiomics features, offers an effective tool for predicting initial recurrence risk in PTC. This approach could significantly aid in clinical decision-making, ultimately contributing to more personalized treatment plans for PTC patients.

## AUTHORS’ CONTRIBUTIONS

The authors confirm their contribution to the paper as follows: F.W.: Conceptualization; Y.X.: Data collection: Y.S.: Data Curation: Y.Z., X.X.: Draft manuscript. All authors reviewed the results and approved the final version of the manuscript.

## Figures and Tables

**Fig. (2):**
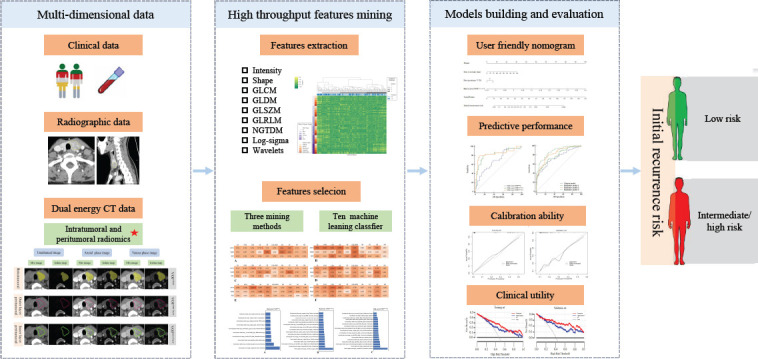
Radiomics workflow.

**Fig. (3):**
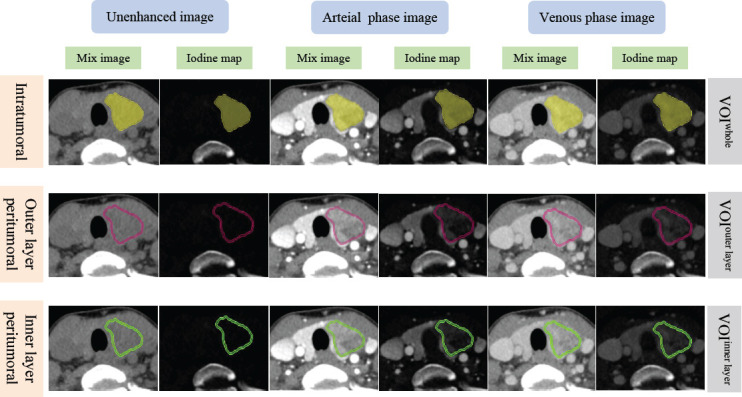
Intratumoral and peritumoral segmentation for radiomics analysis. Semi-automatic segmentation of the intratumoral tumor region (VOI^whole^) was first conducted by a radiologist using the software (yellow region). With the help of the object operations and combination module integrated in the software, the peritumoral region with 1 mm outer (VOI^outer layer^, pink region) and inner (VOI^inner layer^, green region) distance to the tumor boundary surface was automatically segmented, respectively.

**Fig. (4):**
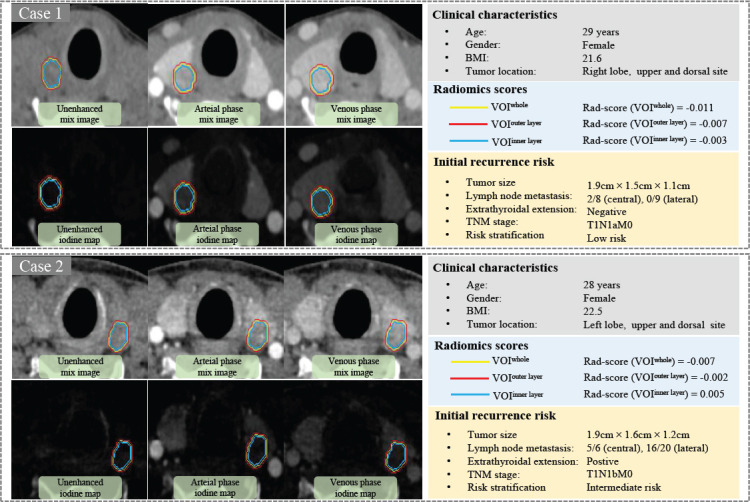
Two presented cases of PTC patients with low and intermediate initial recurrence risk, demonstrating similar clinical characteristics but significantly different rad-score (VOI^whole^) (−0.011 *vs*. -0.007), rad-score (VOI^outer layer^) (−0.007 *vs*. -0.002), and rad-score (VOI^inner layer^) (−0.003 *vs*. 0.005).

**Fig. (5):**
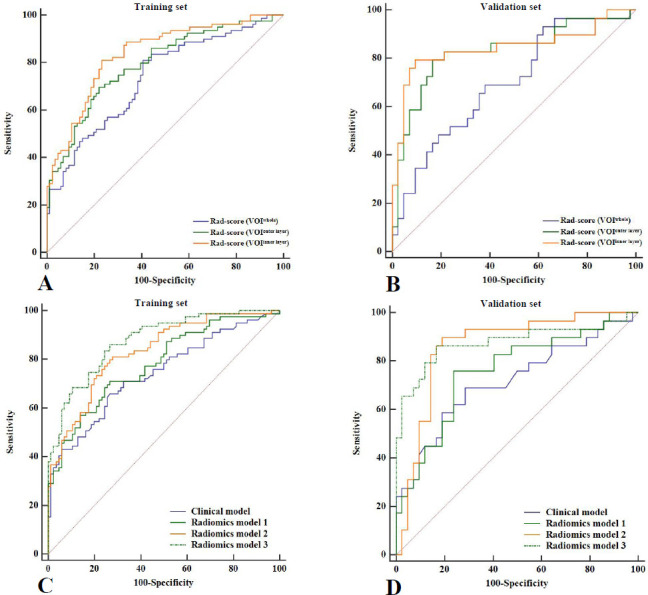
Performances of different rad-scores and models for predicting initial recurrence risk in PTC patients. The peritumoral rad-scores (VOI^outer layer^, VOI^inner layer^) significantly outperformed the intratumoral rad-score (VOI^whole^) in both the training (**A**) and validation set (**B**). All three radiomics models performed significantly better than the clinical model, with radiomics model 3 achieving the best performance in both the training (**C**) and validation (**D**) sets.

**Table 1:** Clinical characteristics of patients with PTC grouped by initial recurrence risk.

**Characteristics**	**Training Set (165)**			**Validation Set (71)**		
	**Low Risk** **(86)**	**Intermediate/High Risk (79)**	**P**	**Low Risk** **(42)**	**Intermediate/High Risk (29)**	**P**
Age (y) *	38 ± 12	37 ± 12	0.732	39 ± 13	37 ± 10	0.340
**Age (y)**	-	-	0.419	-	-	0.271
**≤55**	75 (87.2%)	72 (91.1%)	-	34 (81.0%)	27 (93.1%)	-
**>55**	11 (12.8%)	7 (8.9%)	-	8 (19.0%)	2 (6.9%)	-
**Age (y)**	-	-	0.616	-	-	0.192
**≤45**	65 (75.6%)	57 (72.2%)	-	29 (69.0%)	24 (82.8%)	
**>45**	21 (24.4%)	22 (27.8%)	-	13 (31.0%)	5 (17.2%)	
**Gender**	-	-	0.298	-	-	0.080
**Male**	24 (27.9%)	28 (35.4%)	-	17 (40.5%)	6 (20.7%)	
**Female**	62 (72.1%)	51 (64.6%)	-	25 (59.5%)	23 (79.3%)	
**BMI (kg/m^2^)**	-	-	0.551	-	-	0.989
**<25**	55 (64.0%)	54 (68.4%)	-	26 (61.9%)	18 (62.1%)	
**≥25**	31 (36.0%)	25 (31.6%)	-	16 (38.1%)	11 (37.9%)	
**Nodular goiter**	-	-	0.798	-	-	0.915
**Negative**	55 (64.0%)	49 (62.0%)	-	27 (64.3%)	19 (65.5%)	
**Positive**	31 (36.0%)	30 (38.0%)	-	15 (35.7%)	10 (34.5%)	
**Hashimoto thyroiditis**	-	-	0.172	-	-	0.489
**Negative**	57 (66.3%)	60 (75.9%)	-	28 (66.7%)	17 (58.6%)	
**Positive**	29 (33.7%)	19 (24.1%)	-	14 (33.3%)	12 (41.4%)	

**Notes:** Data are numbers of patients and parentheses indicate the proportion if not specified. PTC, papillary thyroid cancer; BMI, body mass index. *Values were reported in mean **±** standard deviation.

**Table 2:** Radiographic characteristics of patients with PTC grouped by initial recurrence risk.

**Characteristics**	**Training Set (165)**			**Validation Set (71)**		
	**Low Risk** **(86)**	**Intermediate/** **High Risk (79)**	**P**	**Low Risk** **(42)**	**Intermediate/** **High Risk (29)**	**P**
Size (axial, mm)*	14.1 ± 4.3	19.9 ± 7.5	<0.001	13.8 ± 3.3	19.8 ± 7.6	<0.001
Size (coronal, mm)*	15.3 ± 4.7	22.8 ± 10.1	<0.001	14.6 ± 2.9	22.2 ± 8.5	<0.001
**Location**	-	-	0.402	-	-	0.586
**Right lobe**	41 (47.7%)	38 (48.1%)	-	22 (52.4%)	16 (55.2%)	-
**Left lobe**	39 (45.3%)	39 (49.4%)	-	19 (45.2%)	11 (37.9%)	-
**Isthmus**	6 (7.0%)	2 (2.5%)	-	1 (2.4%)	2 (6.9%)	-
**Site (position, S-I)**	-	-	0.251	-	-	0.420
**Superior**	24 (27.9%)	21 (26.6%)	-	15 (35.7%)	13 (44.8%)	-
**Medium**	35 (40.7%)	24 (30.4%)	-	14 (33.3%)	11 (37.9%)	-
**Inferior**	27 (31.4%)	34 (43.0%)	-	13 (31.0%)	5 (17.2%)	-
**Site (position, V-D)**	-	-	0.042	-	-	0.017
**Ventral**	31 (36.0%)	21 (26.6%)	-	13 (31.0%)	4 (13.8%)	-
**Medium**	27 (31.4%)	40 (50.6%)	-	16 (38.1%)	21 (72.4%)	-
**Dorsal**	28 (32.6%)	18 (22.8%)	-	13 (31.0%)	4 (13.8%)	-
**Aspect ratio**	-	-	0.733	-	-	0.796
**≤1 (wider than tall)**	15 (17.4%)	10 (12.7%)	-	8 (19.0%)	4 (13.8%)	-
**>1 (taller than wide)**	71 (82.6%)	69 (87.3%)	-	34 (81.0%)	25 (86.2%)	-
**Shape**	-	-	0.417	-	-	0.678
**Regular**	22 (25.6%)	16 (20.3%)	-	12 (28.6%)	7 (24.1%)	-
**Irregular**	64 (74.4%)	63 (79.7%)	-	30 (71.4%)	22 (75.9%)	-
**Calcification**	-	-	0.750	-	-	0.522
**No calcification**	60 (69.8%)	51 (64.5%)	-	31 (73.8%)	18 (62.1%)	-
**Macrocalcification**	13 (15.1%)	13 (16.5%)	-	5 (11.9%)	4 (13.8%)	-
**Microcalcification**	13 (15.1%)	15 (19.0%)	-	6 (14.3%)	7 (24.1%)	-
**Cystic**	-	-	0.287	-	-	0.999
**Negative**	73 (84.9%)	62 (78.5%)	-	37 (88.1%)	25 (86.2%)	-
**Positive**	13 (15.1%)	17 (21.5%)	-	5 (11.9%)	4 (13.8%)	-
**Rad-score (VOI^whole^)^†^**	-0.040 (-0.057, -0.008)	-0.002 (-0.029, 0.086)	<0.001	-0.041 (-0.058, -0.002)	-0.013 (-0.044, 0.039)	0.020
**Rad-score (VOI^outer layer^)^†^**	-0.045 (-0.076, -0.011)	0.033 (-0.018, 0.139)	<0.001	-0.050 (-0.070, 0.006)	0.002 (-0.034, 0.076)	0.003
**Rad-score (VOI^inner layer^)^†^**	-0.038 (-0.067, -0.002)	0.047 (0.007, 0.124)	<0.001	-0.048 (-0.066, 0.029)	0.015 (-0.014, 0.072)	0.004

**Notes:** Data are numbers of patients and parentheses indicate the proportion if not specified. PTC, papillary thyroid cancer; VOI, volume of interest. * Values were reported in mean ± standard deviation. ^†^ Data are medians, with interquartile range in parentheses.

**Table 3:** Results of multivariate logistic analysis to establish clinical and radiomics models.

**Models**	**β**	**Odds Ratio (95% CI)**	**P**	**VIF**
Clinical model	-	-	-	-
Size (coronal, mm)	0.351	1.420 (1.174, 1.718)	<0.001	1.014
Site (position, V-D)	0.465	1.592 (0.659, 3.845)	0.030	1.014
Radiomics model 1	-	-	-	-
Size (coronal, mm)	0.320	1.377 (1.138, 1.666)	0.001	1.081
Site (position, V-D)	0.443	1.558 (0.644, 3.770)	0.036	1.014
Rad-score (VOI^whole^)	0.356	1.428 (0.702, 2.903)	0.035	1.067
Radiomics model 2	-	-	-	-
Size (coronal, mm)	0.298	1.347 (1.123, 1.616)	0.001	1.172
Site (position, V-D)	0.506	1.659 (0.627, 4.391)	0.031	1.014
Rad-score (VOI^outer layer^)	1.157	3.180 (1.448, 6.985)	0.004	1.156
Radiomics model 3	-	-	-	-
Size (coronal, mm)	0.247	1.281 (1.044, 1.570)	0.017	1.286
Site (position, V-D)	0.384	1.469 (0.573, 3.764)	0.042	1.015
Rad-score (VOI^inner layer^)	1.302	3.677 (1.516, 8.920)	0.004	1.269

**Notes:** Numbers in the parentheses are the 95% confidence interval. CI, confidence interval; VIF, variance inflation factor; VOI, volume of interest.

**Table 4:** Performances of different models for predicting initial recurrence risk in patients with PTC.

**-**	**AUC**	**Sensitivity**	**Specificity**	**PPV**	**NPV**
Training set	-	-	-	-	-
Clinical model	0.741 (0.667, 0.806)	0.658 (0.543, 0.761)	0.733 (0.626, 0.822)	0.693 (0.576, 0.795)	0.700 (0.594, 0.792)
Radiomics model 1	0.780 (0.709, 0.841)	0.709 (0.596, 0.806)	0.733 (0.626, 0.822)	0.709 (0.596, 0.806)	0.733 (0.626, 0.822)
Radiomics model 2	0.831 (0.765, 0.885)	0.810 (0.706, 0.890)	0.721 (0.614, 0.812)	0.727 (0.622, 0.817)	0.805 (0.699, 0.887)
Radiomics model 3	0.877 (0.817, 0.923)	0.861 (0.765, 0.928)	0.733 (0.626, 0.822)	0.747 (0.645, 0.833)	0.851 (0.750, 0.923)
Validation set	-	-	-	-	-
Clinical model	0.715 (0.596, 0.816)	0.690 (0.492, 0.847)	0.714 (0.554, 0.843)	0.625 (0.437, 0.789)	0.769 (0.607, 0.889)
Radiomics model 1	0.758 (0.642, 0.852)	0.759 (0.565, 0.897)	0.762 (0.605, 0.879)	0.687 (0.500, 0.839)	0.821 (0.665, 0.925)
Radiomics model 2	0.860 (0.758, 0.931)	0.897 (0.726, 0.978)	0.810 (0.659, 0.914)	0.765 (0.588, 0.893)	0.919 (0.781, 0.983)
Radiomics model 3	0.876 (0.776, 0.942)	0.862 (0.683, 0.961)	0.833 (0.686, 0.930)	0.781 (0.600, 0.907)	0.897 (0.758, 0.971)

**Notes:** Numbers in the parentheses are the 95% confidence interval. PTC, papillary thyroid cancer, AUC, area under curve, PPV, positive predictive value, NPV, negative predictive value.

## Data Availability

All data generated or analyzed during this study are included in this published article.

## LIST OF ABBREVIATIONS

PTC: Papillary Thyroid Cancer
ATA: American Thyroid Association
CT: Computed Tomography
DECT: Dual-Energy CT
BMI: Body Mass Index
VOIs: Volumes of Interest
FAE: Feature Explorer
ICC: Intraclass Correlation Coefficient
VIF: Variance Inflation Factor
ANOVA: Analysis of Variance
AE: Auto-Encoder
LDA: Linear Discriminant Analysis
AUC: Area Under the Curve
ROC: Receiver Operating Characteristic Curve

## STANDARDS OF REPORTING

STROBE guidelines were followed.

## References

[1] Haugen B.R., Alexander E.K., Bible K.C., Doherty G.M., Mandel S.J., Nikiforov Y.E., Pacini F., Randolph G.W., Sawka A.M., Schlumberger M., Schuff K.G., Sherman S.I., Sosa J.A., Steward D.L., Tuttle R.M., Wartofsky L. (2016). 2015 american thyroid association management guidelines for adult patients with thyroid nodules and differentiated thyroid cancer: The american thyroid association guidelines task force on thyroid nodules and differentiated thyroid cancer.. Thyroid.

[2] Tuttle R.M., Alzahrani A.S. (2019). Risk stratification in differentiated thyroid cancer: From detection to final follow-up.. J. Clin. Endocrinol. Metab..

[3] Xu S., Huang H., Qian J., Liu Y., Huang Y., Wang X., Liu S., Xu Z., Liu J. (2021). Prevalence of hashimoto thyroiditis in adults with papillary thyroid cancer and its association with cancer recurrence and outcomes.. JAMA Netw. Open.

[4] Gajowiec A., Chromik A., Furga K., Skuza A., Gąsior-Perczak D., Walczyk A., Pałyga I., Trybek T., Mikina E., Szymonek M., Gadawska-Juszczyk K., Kuchareczko A., Suligowska A., Jaskulski J., Orłowski P., Chrapek M., Góźdź S., Kowalska A. (2021). Is male sex a prognostic factor in papillary thyroid cancer?. J. Clin. Med..

[5] Chatchomchuan W., Thewjitcharoen Y., Karndumri K., Porramatikul S., Krittiyawong S., Wanothayaroj E., Vongterapak S., Butadej S., Veerasomboonsin V., Kanchanapitak A., Rajatanavin R., Himathongkam T. (2021). Recurrence factors and characteristic trends of papillary thyroid cancer over three decades.. Int. J. Endocrinol..

[6] Kuo C.Y., Yang P.S., Chien M.N., Cheng S.P. (2020). Preoperative factors associated with extrathyroidal extension in papillary thyroid cancer.. Eur. Thyroid J..

[7] Kim S.Y., Kwak J.Y., Kim E.K., Yoon J.H., Moon H.J. (2015). Association of preoperative us features and recurrence in patients with classic papillary thyroid carcinoma.. Radiology.

[8] Nam S.Y., Shin J.H., Han B.K., Ko E.Y., Ko E.S., Hahn S.Y., Chung J.H. (2013). Preoperative ultrasonographic features of papillary thyroid carcinoma predict biological behavior.. J. Clin. Endocrinol. Metab..

[9] Mayerhoefer M.E., Materka A., Langs G., Häggström I., Szczypiński P., Gibbs P., Cook G. (2020). Introduction to radiomics.. J. Nucl. Med..

[10] Tepe M., Sevin E., Inan I., Aktan A., Ayaz M., Ibrahim Ali H., Senturk S. (2024). Utilizing CT and MRI in assessing peritumoral neovascularization in renal cell carcinoma: A comprehensive analysis of histological subtypes and tumor characteristics by imaging.. Curr. Med. Imaging.

[11] O’Connor J.P.B., Rose C.J., Waterton J.C., Carano R.A.D., Parker G.J.M., Jackson A. (2015). Imaging intratumor heterogeneity: Role in therapy response, resistance, and clinical outcome.. Clin. Cancer Res..

[12] Alabousi M., Alabousi A., Adham S., Pozdnyakov A., Ramadan S., Chaudhari H., Young J.E.M., Gupta M., Harish S. (2022). Diagnostic test accuracy of ultrasonography *vs* computed tomography for papillary thyroid cancer cervical lymph node metastasis.. JAMA Otolaryngol. Head Neck Surg..

[13] Lee J.H., Ha E.J., Kim D., Jung Y.J., Heo S., Jang Y., An S.H., Lee K. (2020). Application of deep learning to the diagnosis of cervical lymph node metastasis from thyroid cancer with CT: External validation and clinical utility for resident training.. Eur. Radiol..

[14] Yu J., Deng Y., Liu T., Zhou J., Jia X., Xiao T., Zhou S., Li J., Guo Y., Wang Y., Zhou J., Chang C. (2020). Lymph node metastasis prediction of papillary thyroid carcinoma based on transfer learning radiomics.. Nat. Commun..

[15] Yu P., Wu X., Li J., Mao N., Zhang H., Zheng G., Han X., Dong L., Che K., Wang Q., Li G., Mou Y., Song X. (2022). Extrathyroidal extension prediction of papillary thyroid cancer with computed tomography based radiomics nomogram: A multicenter study.. Front. Endocrinol..

[16] Meng XW, Pi YW, Wang GL, Qi SN, Zhang GH, Cheng YX (2024). The relationship between quantitative parameters of dual-energy CT and HIF-1α expression in non-small cell lung cancer.. Curr. Med. Imaging.

[17] Hamid S., Nasir M.U., So A., Andrews G., Nicolaou S., Qamar S.R. (2021). Clinical applications of dual-energy CT.. Korean J. Radiol..

[18] Agrawal M.D., Pinho D.F., Kulkarni N.M., Hahn P.F., Guimaraes A.R., Sahani D.V. (2014). Oncologic applications of dual-energy CT in the abdomen.. Radiographics.

[19] Zhou Y., Su G.Y., Hu H., Tao X.W., Ge Y.Q., Si Y., Shen M.P., Xu X.Q., Wu F.Y. (2022). Radiomics from primary tumor on dual-energy CT derived iodine maps can predict cervical lymph node metastasis in papillary thyroid cancer.. Acad. Radiol..

[20] Xu X.Q., Zhou Y., Su G.Y., Tao X.W., Ge Y.Q., Si Y., Shen M.P., Wu F.Y. (2022). Iodine maps from dual-energy CT to predict extrathyroidal extension and recurrence in papillary thyroid cancer based on a radiomics approach.. AJNR Am. J. Neuroradiol..

[21] Xu X., Zhang H.L., Liu Q.P., Sun S.W., Zhang J., Zhu F.P., Yang G., Yan X., Zhang Y.D., Liu X.S. (2019). Radiomic analysis of contrast-enhanced CT predicts microvascular invasion and outcome in hepatocellular carcinoma.. J. Hepatol..

[22] Hu Y., Xie C., Yang H., Ho J.W.K., Wen J., Han L., Chiu K.W.H., Fu J., Vardhanabhuti V. (2020). Assessment of intratumoral and peritumoral computed tomography radiomics for predicting pathological complete response to neoadjuvant chemoradiation in patients with esophageal squamous cell carcinoma.. JAMA Netw. Open.

[23] Zhou Y., Gu H.L., Zhang X.L., Tian Z.F., Xu X.Q., Tang W.W. (2022). Multiparametric magnetic resonance imaging-derived radiomics for the prediction of disease-free survival in early-stage squamous cervical cancer.. Eur. Radiol..

[24] Dewaguet J., Copin M.C., Duhamel A., Faivre J.B., Deken V., Sedlmair M., Flohr T., Schmidt B., Cortot A., Wasielewski E., Remy J., Remy-Jardin M. (2022). Dual-energy CT perfusion of invasive tumor front in non–small cell lung cancers.. Radiology.

[25] Wels M.G., Lades F., Muehlberg A., Suehling M. (2019). General purpose radiomics for multi-modal clinical research.. Proc. SPIE.

[26] Moltz J.H., Bornemann L., Kuhnigk J.M., Dicken V., Peitgen E., Meier S., Bolte H., Fabel M., Bauknecht H-C., Hittinger M., Kießling A., Pusken M., Peitgen H-O. (2009). Advanced segmentation techniques for lung nodules, liver metastases, and enlarged lymph nodes in CT scans.. IEEE J. Sel. Top. Signal Process..

[27] van Griethuysen J.J.M., Fedorov A., Parmar C., Hosny A., Aucoin N., Narayan V., Beets-Tan R.G.H., Fillion-Robin J.C., Pieper S., Aerts H.J.W.L. (2017). Computational radiomics system to decode the radiographic phenotype.. Cancer Res..

[28] Song Y., Zhang J., Zhang Y., Hou Y., Yan X., Wang Y., Zhou M., Yao Y., Yang G. (2020). FeAture explorer (FAE): A tool for developing and comparing radiomics models.. PLoS One.

[29] Umirzakova S., Muksimova S., Baltayev J., Cho Y.I. (2025). Force map-enhanced segmentation of a lightweight model for the early detection of cervical cancer.. Diagnostics.

[30] Zhang Z. (2016). Variable selection with stepwise and best subset approaches.. Ann. Transl. Med..

[31] O’brien R.M. (2007). A caution regarding rules of thumb for variance inflation factors.. Qual. Quant..

[32] Kramer A.A., Zimmerman J.E. (2007). Assessing the calibration of mortality benchmarks in critical care: The Hosmer-Lemeshow test revisited.. Crit. Care Med..

[33] Fitzgerald M., Saville B.R., Lewis R.J. (2015). Decision curve analysis.. JAMA.

[34] Xu H., Wu W., Zhao Y., Liu Z., Bao D., Li L., Lin M., Zhang Y., Zhao X., Luo D. (2023). Analysis of preoperative computed tomography radiomics and clinical factors for predicting postsurgical recurrence of papillary thyroid carcinoma.. Cancer Imaging.

