# Supplementary material for: Initial Recurrence Risk Stratification of Papillary Thyroid Cancer based on Intratumoral and Peritumoral Dual Energy CT Radiomics
Source: Curr Med Imaging. 2025 Aug 21;21:e15734056402179. doi: 10.2174/0115734056402179250813050300 (PMC13223471; doi:10.2174/0115734056402179250813050300)
Supplement: Supplementary file 1 [file CMIM-21-E15734056402179_SD1.pdf]

1573-4056/25 *Send Orders for Reprints to reprints@benthamscience.net*

BENTHAM SCIENCE
CrossMark

Current Medical Imaging

Content list available at: https://benthamscience.com/journals/cmir

Supplementary Material

Initial Recurrence Risk Stratification of Papillary Thyroid Cancer based on Intratumoral and Peritumoral Dual Energy CT Radiomics

Yan Zhou[1], Yongkang Xu[1], Yan Si[2], Feiyun Wu[1] and Xiaoquan Xu[1,*]

[1]*Department of Radiology, The First Affiliated Hospital with Nanjing Medical University, Nanjing, China*
[2]*Department of Thyroid Surgery, The First Affiliated Hospital with Nanjing Medical University, Nanjing, China*

1. Supplementary S1. DECT Scanning Protocol and Post-Processing

All DECT examinations were performed with a third-generation DECT scanner (Somatom Force; Siemens Healthcare, Erlangen, Germany) equipped with two X-ray tube voltages. DECT scanning protocol was listed as follows: detector collimation, 128 × 0.6 mm; rotation time, 0.5 sec; pitch factor, 0.7; matrix, 256 × 256; field of view, 252 × 252 mm. After unenhanced CT scan, 75 mL of contrast agent (iopromide; Bayer HealthCare, Berlin, Germany) was administrated through cubital vein at a flow rate of 3.5 mL/sec, followed by a scan time for arterial phase of 25 sec and venous phase of 50 sec. To reduce radiation dose, real-time automatic tube current modulation (CARE dose 4D; Siemens Healthcare) was applied. The mean volume CT dose index (CTDIvol) and dose-length product (DLP) were 14.2 ± 2.1 mGy (range, 9.3-18.1 mGy) and 397.5 ± 58.5 mGy × cm (range, 260.2-506.8 mGy × cm), respectively.

All DECT image reconstruction was conducted on a commercially available workstation with application of Syngo Dual Energy (Siemens Healthcare). After each neck DECT scanning, a slice thickness of 1.5 mm of three different image sets were created by a dual-energy system, including 80 kVp, Sn150 kVp and a weighted average image (mix image) that was calculated from 80 kVp and Sn150 kVp tube data at a ratio of 0.5:0.5, which is typically regarded equivalent to the conventional 120 kVp single-energy CT. Iodine quantification was performed using quantitative soft tissue kernel (Q40), based on the three-material decomposition algorithm integrated in the software. Iodine maps reflect the content and distribution of tissue contrast agent (iodine) at a 1.5 mm slice thickness can be autogenerated.

Table S1. Clinicopathological characteristics of our study cohort.

Characteristics	Training set (165)	Validation set (71)	P
**T stage**	-	-	0.989
**T1**	120 (72.7%)	53 (74.6%)	-
**T2**	32 (19.4%)	13 (18.3%)	-
**T3**	10 (6.1%)	4 (5.6%)	-
**T4**	3 (1.8%)	1 (1.4%)	-
**N stage**	-	-	0.394
**N0**	32 (19.4%)	14 (19.7%)	-
**N1a**	58 (35.2%)	31 (43.7%)	-
**N1b**	75 (45.5%)	26 (36.6%)	-
**M stage**	-	-	0.999
**M0**	164 (99.4%)	71 (100.0%)	-
**M1**	1 (0.6%)	0 (0.0%)	-
**Initial recurrence risk**	-	-	0.467
**Low risk**	86 (52.1%)	42 (59.2%)	-
**Intermediate risk**	54 (32.7%)	22 (31.0%)	-

DOI: 10.2174/0115734056402179250813050300, 2025, *21*, e15734056402179

Characteristics	Training set (165)	Validation set (71)	P
**High risk**	25 (15.2%)	7 (9.9%)	-

Note: Data are numbers of patients and parentheses indicate the proportion.

Table S2. Clinical characteristics of patients in the training and validation sets.

Characteristics	Training set (165)	Validation set (71)	P
**Age (y)***	38 ± 12	38 ± 12	0.808
**Age (y)**	-	-	0.489
**≤55**	147 (89.1%)	61 (85.9%)	-
**>55**	18 (10.9%)	10 (14.1%)	-
**Age (y)**	-	-	0.909
**≤45**	122 (73.9%)	53 (74.6%)	-
**>45**	43 (26.1%)	18 (25.4%)	-
**Gender**	-	-	0.894
**Male**	52 (31.5%)	23 (32.4%)	-
**Female**	113 (68.5%)	48 (67.6%)	-
**BMI (kg/m^2)**	-	-	0.546
**<25**	109 (66.1%)	44 (62.0%)	-
**≥25**	56 (33.9%)	27 (38.0%)	-
**Nodular goiter**	-	-	0.797
**Negative**	104 (63.0%)	46 (64.8%)	-
**Positive**	61 (37.0%)	25 (35.2%)	-
**Hashimoto thyroiditis**	-	-	0.253
**Negative**	117 (70.9%)	45 (63.4%)	-
**Positive**	48 (29.1%)	26 (36.6%)	-

Data are numbers of patients and parentheses indicate the proportion if not specified. BMI, body mass index.
*Values were reported in mean ±standard deviation.

Table S3. Radiographic characteristics of patients in the training and validation sets

Characteristics	Training set (165)	Validation set (71)	P
**Size (axial, mm)***	16.8 ± 6.7	16.2 ± 6.2	0.531
**Size (coronal, mm)***	18.9 ± 8.7	17.7 ± 6.9	0.262
**Location**	-	-	0.729
**Right lobe**	79 (47.9%)	38 (53.5%)	-
**Left lobe**	78 (47.2%)	30 (42.3%)	-
**Isthmus**	8 (4.8%)	3 (4.2%)	-
**Site (position, S-I)**	-	-	0.099
**Superior**	45 (27.3%)	28 (39.4%)	-
**Medium**	59 (35.8%)	25 (35.2%)	-
**Inferior**	61 (37.0%)	18 (25.4%)	-
**Site (position, V-D)**	-	-	0.272
**Ventral**	52 (31.5%)	17 (23.9%)	-
**Medium**	67 (40.6%)	37 (52.1%)	-
**Dorsal**	46 (27.9%)	17 (23.9%)	-
**Aspect ratio**	-	-	0.735
**≤1 (wider than tall)**	25 (15.2%)	12 (16.9%)	-
**>1 (taller than wide)**	140 (84.8%)	59 (83.1%)	-
**Shape**	-	-	0.539
**Regular**	38 (23.0%)	19 (26.8%)	-
**Irregular**	127 (77.0%)	52 (73.2%)	-
**Calcification**	-	-	0.822
**No calcification**	111 (67.3%)	49 (69.0%)	-
**Macrocalcification**	26 (15.8%)	9 (12.7%)	-

Characteristics	Training set (165)	Validation set (71)	P
**Microcalcification**	28 (17.0%)	13 (18.3%)	-
**Cystic**	-	-	0.296
**Negative**	135 (81.8%)	62 (87.3%)	-
**Positive**	30 (18.2%)	9 (12.7%)	-
**Rad-score (VOI^{whole})†**	-0.022 (-0.047, 0.016)	-0.031 (-0.056, 0.016)	0.479
**Rad-score ($VOI^{outer\ layer}$)†**	-0.013 (-0.057, 0.044)	-0.019 (-0.059, 0.043)	0.552
**Rad-score ($VOI^{inner\ layer}$)†**	0.003 (-0.048, 0.059)	-0.003 (-0.057, 0.048)	0.230

Data are numbers of patients and parentheses indicate the proportion if not specified. VOI, volume of interest.
* Values were reported in mean ± standard deviation.
†Data are medians, with interquartile range in parentheses.

Table S4. Predictive performances of combinations of different machine learning algorithms for building three types of rad-scores.

-	LR	SVM	LDA	AE	RF	LRLASSO	AB	DT	GP	NB
**Training set**	-	-	-	-	-	-	-	-	-	-
**Rad-score (VOI^{whole})**	-	-	-	-	-	-	-	-	-	-
**ANOVA**	0.735	0.720	0.734	0.735	0.718	0.735	0.813	0.815	0.734	0.675
**Relief**	0.703	0.697	0.704	0.525	0.874	0.528	0.863	0.809	0.712	0.718
**RFE**	0.735	0.720	0.734	0.726	0.729	0.734	0.714	0.728	0.734	0.718
**Rad-score ($VOI^{outer\ layer}$)**	-	-	-	-	-	-	-	-	-	-
**ANOVA**	0.775	0.782	0.794	0.780	0.773	0.761	0.781	0.774	0.772	0.778
**Relief**	0.780	0.781	0.777	0.814	0.809	0.739	0.785	0.833	0.785	0.778
**RFE**	0.780	0.739	0.780	0.788	0.729	0.760	0.780	0.765	0.780	0.777
**Rad-score ($VOI^{inner\ layer}$)**	-	-	-	-	-	-	-	-	-	-
**ANOVA**	0.795	0.798	0.837	0.804	0.814	0.766	0.783	0.803	0.793	0.785
**Relief**	0.741	0.739	0.792	0.808	0.811	0.739	0.780	0.799	0.785	0.786
**RFE**	0.750	0.718	0.777	0.752	0.794	0.760	0.772	0.767	0.700	0.746
**Validation set**	-	-	-	-	-	-	-	-	-	-
**Rad-score (VOI^{whole})**	-	-	-	-	-	-	-	-	-	-
**ANOVA**	0.691	0.694	0.691	0.696	0.623	0.691	0.509	0.601	0.661	0.522
**Relief**	0.544	0.567	0.567	0.653	0.505	0.697	0.540	0.514	0.531	0.539
**RFE**	0.631	0.614	0.691	0.640	0.652	0.691	0.659	0.619	0.626	0.522
**Rad-score ($VOI^{outer\ layer}$)**	-	-	-	-	-	-	-	-	-	-
**ANOVA**	0.731	0.720	0.827	0.808	0.619	0.731	0.666	0.634	0.730	0.672
**Relief**	0.727	0.627	0.774	0.679	0.593	0.732	0.656	0.563	0.815	0.674
**RFE**	0.637	0.632	0.786	0.734	0.672	0.731	0.666	0.634	0.759	0.675

-	LR	SVM	LDA	AE	RF	LRLASSO	AB	DT	GP	NB
**Rad-score (VOI$^{inner\ layer}$)**	-	-	-	-	-	-	-	-	-	-
**ANOVA**	0.748	0.788	0.843	0.808	0.733	0.709	0.697	0.702	0.769	0.698
**Relief**	0.731	0.751	0.794	0.696	0.602	0.752	0.642	0.702	0.774	0.664
**RFE**	0.709	0.713	0.785	0.710	0.725	0.728	0.676	0.689	0.762	0.678

Note: Data are area under curves of using combinations of different machine learning algorithms. VOI, volume of interest; ANOVA, analysis of variance; RFE, recursive feature elimination; LR, logistic regression; SVM, support vector machine; LDA, linear discriminant analysis; AE, auto-encoder; RF, random forest; LRLASSO, logistic regression via lasso; AB, ada-boost; DT, decision tree; GP, gaussian process; NB, native bayes.

Table S5. Biological and clinical interpretation of final selected single radiomics feature in three different types of rad-scores.

Sequence	Features	Biological and clinical interpretation
**Rad-score (VOI^{whole})**	Unenhanced iodine map_original_firstorder_Entropy	Measures the disorder in the image's gray level distribution. Higher entropy indicates greater tissue heterogeneity, which could be linked to more aggressive tumor behavior
	Unenhanced mix image_original_shape_Compactness2	Describes the compactness of the tumor shape. Lower values suggest irregular tumor morphology, which is often associated with higher invasiveness
	Arterial phase iodine map_original_firstorder_90Percentile	The 90th percentile of pixel intensity in the arterial phase, reflecting the level of blood supply. High values suggest a tumor with increased vascularity, often linked to rapid growth
	Arterial phase iodine map_original_glcm_ClusterTendency	Measures how similar pixels group together. A higher clustering tendency indicates more homogeneous tissue, which may suggest a more stable, less aggressive tumor
	Arterial phase iodine map_wavelet.HLL_glszm_GrayLevelNonUniformity	This texture feature measures gray level variations. High non-uniformity indicates increased tumor heterogeneity, which could be associated with more aggressive or advanced stages of cancer
	Arterial phase iodine map _wavelet.LHH_glcm_DifferenceEntropy	Evaluates the entropy in the gray level co-occurrence matrix. Higher values indicate increased complexity and irregularities in the tumor’s structure, possibly reflecting higher aggressiveness
	Arterial phase mix image_wavelet.LLH_gldm_DependenceVariance	Measures the variance of the dependence matrix, reflecting how gray levels are distributed in relation to each other. High variance suggests more heterogeneity, which may be linked to tumor invasiveness
	Venous phase iodine map_logarithm_glrlm_RunEntropy	Measures the complexity of the gray-level run-length matrix. High entropy reflects more complex textures, indicating greater tumor heterogeneity, often linked to higher malignancy
	Venous phase iodine map_wavelet.LLH_gldm_GrayLevelVariance	Quantifies the variance of gray levels. Higher variance indicates more complex and heterogeneous tissue, potentially suggesting a more aggressive tumor phenotype
	Venous phase mix image_original_firstorder_Skewness	Measures the asymmetry in the intensity distribution of the image. Positive skewness suggests a concentration of high-intensity pixels, potentially indicating aggressive tumor regions

Sequence	Features	Biological and clinical interpretation
Rad-score (VOI$^{outer\ layer}$)	Unenhanced iodine map_original_shape_MajorAxisLength	Represents the largest dimension of the tumor. Longer axis lengths often correlate with larger, more advanced tumors, which may have higher malignant potential
	Unenhanced mix image_wavelet.HHL_firstorder_Entropy	Measures the entropy in wavelet-transformed images. High entropy may indicate more complex and heterogeneous tissue structures, often associated with aggressive tumor behavior
	Unenhanced mix image_wavelet.LLH_glcm_Idn	Assesses the uniformity of pixel pairs in the gray level co-occurrence matrix. Higher values suggest more homogeneous tissue, which may be associated with less aggressive tumors
	Arterial phase iodine map_original_firstorder_Kurtosis	Evaluates the peakedness of the intensity distribution. Higher kurtosis suggests a more pronounced concentration of certain intensities, often indicating areas of high vascularity in tumors
	Arterial phase iodine map_logarithm_glcm_DifferenceAverage	Measures the average difference between pixel pairs in the gray level co-occurrence matrix. Higher values indicate more variability in tissue structure, often linked to malignancy
	Arterial phase iodine map_wavelet.HHH_gldm_LowGrayLevelEmphasis	Reflects the emphasis on low gray levels in the gray-level dependence matrix. High emphasis could indicate regions of necrosis or less active tumor regions
	Arterial phase iodine map_wavelet.LHL_glcm_JointEnergy	Measures the energy in the joint gray level co-occurrence matrix. Higher values suggest a more homogeneous and organized tissue structure, often seen in less aggressive tumors
	Arterial phase iodine map_wavelet.LLH_ngtdm_Complexity	Assesses the complexity of the tumor texture. Higher complexity suggests increased tumor heterogeneity, which is often associated with more aggressive tumors
	Arterial phase iodine map_square_glrlm_GrayLevelNonUniformity	Measures the non-uniformity in the gray-level run-length matrix. Greater non-uniformity typically reflects tumor heterogeneity and aggressiveness
	Arterial phase mix image_wavelet.HLH_firstorder_TotalEnergy	Quantifies the total energy in the image, which correlates with the intensity and texture of the tumor. Higher energy values often suggest tumors with high metabolic activity
	Arterial phase mix image_wavelet.LHH_glrlm_GrayLevelVariance	Measures the variance of the run-lengths in the gray-level run-length matrix. Greater variance often reflects increased tumor complexity and heterogeneity
	Venous phase iodine map_original_glszm_ZonePercentage	Quantifies the percentage of the image covered by different gray-level zones. High values may reflect heterogeneous tumor regions, which are often linked to more aggressive tumors
	Venous phase iodine map_wavelet.HLH_glcm_Idm	Measures the inverse difference moment in the gray-level co-occurrence matrix. High values suggest tissue uniformity, which may indicate less aggressive tumor behavior
	Venous phase iodine map_wavelet.HLL_glrlm_HighGrayLevelRunEmphasis	Emphasizes the presence of long runs of high gray levels. This could indicate areas of necrosis or lower tumor activity
	Venous phase mix image_exponential_firstorder_TotalEnergy	Quantifies the total energy in the image, reflecting overall intensity. Higher values suggest tumors with more active or aggressive growth
	Venous phase mix image_wavelet.LLL_glcm_Idm	Measures the inverse difference moment in the gray-level co-occurrence matrix. Similar to the previous IDM, higher values indicate uniform tissue, typically found in less aggressive tumors

Sequence	Features	Biological and clinical interpretation
	Unenhanced iodine map_wavelet.LLH_glcm_DifferenceAverage	Measures the average difference in pixel intensities within the gray-level co-occurrence matrix. Higher values suggest more variation in the tissue structure, which can be associated with malignancy
	Unenhanced mix image_original_shape_Flatness	Describes the flatness of the tumor's shape. Lower flatness values typically suggest more irregular and invasive tumors
	Arterial phase iodine map_original_gldm_DependenceEntropy	Quantifies the entropy in the gray-level dependence matrix, reflecting the complexity of the tissue. Higher values suggest increased tumor complexity and possible malignancy
	Arterial phase iodine map_logarithm_glcm_SumAverage	Measures the sum of the pixel intensities in the gray-level co-occurrence matrix. Higher values suggest tumors with increased intensity or metabolic activity
	Arterial phase iodine map_logarithm_glszm_ZoneEntropy	Measures entropy in the gray-level zone-size matrix. High entropy may indicate more complex and heterogeneous tumor structures
	Arterial phase iodine map_wavelet.HLH_firstorder_Uniformity	Assesses the uniformity of intensity distribution. Higher uniformity suggests a more organized tissue structure, often seen in less aggressive tumors
	Arterial phase iodine map_wavelet.LHL_glcm_JointEnergy	Measures joint energy in the gray-level co-occurrence matrix. Higher energy indicates more uniform tissue, which is often associated with less aggressive tumors
	Arterial phase iodine map_log.sigma.3.0.mm.3D_gldm_LowGrayLevelEmphasis	Emphasizes low gray levels in the 3D gray-level dependence matrix. High values may indicate necrotic or less active areas within the tumor
**Rad-score (VOI$^{inner\ layer}$)**	Arterial phase mix image_original_glrlm_GrayLevelNonUniformity	Measures non-uniformity in the gray-level run-length matrix. Greater non-uniformity reflects increased tumor heterogeneity and invasiveness
	Arterial phase mix image_squareroot_gldm_LowGrayLevelEmphasis	Similar to previous low gray level emphasis features, this reflects less active tumor areas or necrotic regions
	Arterial phase mix image_wavelet.HLH_gldm_LowGrayLevelEmphasis	Highlights low gray levels in the gray-level dependence matrix. High emphasis may suggest necrotic tissue or less aggressive tumor areas
	Venous phase iodine map_original_glcm_Id	Measures the inverse difference in the gray-level co-occurrence matrix. Higher values indicate more uniform tissue, often linked to less aggressive tumors
	Venous phase iodine map_square_firstorder_Energy	Quantifies the total energy in the image. Higher values indicate greater metabolic activity, which could be linked to more aggressive tumors
	Venous phase iodine map_logarithm_glszm_GrayLevelVariance	Measures the variance in the gray-level zone-size matrix. Higher values indicate more complex and heterogeneous tumor regions, often associated with malignancy
	Venous phase iodine map_wavelet.HLH_glrlm_HighGrayLevelRunEmphasis	Emphasizes long runs of high gray levels, which may indicate necrotic tissue or lower tumor activity
	Venous phase mix image_original_glcm_Imc1	Measures a component of the gray-level co-occurrence matrix, reflecting the uniformity of pixel pairs. Higher values may suggest more homogeneous tumor tissue
	Venous phase mix image_wavelet.HLL_firstorder_Kurtosis	Quantifies the peakedness of the pixel intensity distribution. Higher kurtosis suggests concentrated high-intensity areas, often reflecting regions of high vascularity or aggressive tumor growth

Table S6. The performance of final selected single radiomics feature in three different types of rad-scores.

Sequence	Features	AUC (95% CI)	
		Training set	Validation set
Rad-score (VOI^{whole})	Unenhanced iodine map_original_firstorder_Entropy	0.542 (0.453, 0.630)	0.585 (0.452, 0.718)
	Unenhanced mix image_original_shape_Compactness2	0.537 (0.450, 0.625)	0.539 (0.412, 0.667)
	Arterial phase iodine map_original_firstorder_90Percentile	0.627 (0.541, 0.713)	0.557 (0.419, 0.696)
	Arterial phase iodine map_original_glcm_ClusterTendency	0.616 (0.528, 0.703)	0.606 (0.468, 0.743)
	Arterial phase iodine map_wavelet.HLL_glszm_GrayLevelNonUniformity	0.584 (0.496, 0.672)	0.632 (0.503, 0.762)
	Arterial phase iodine map _wavelet.LHH_glcm_DifferenceEntropy	0.519 (0.432, 0.607)	0.645 (0.510, 0.780)
	Arterial phase mix image_wavelet.LLH_gldm_DependenceVariance	0.523 (0.435, 0.610)	0.562 (0.424, 0.699)
	Venous phase iodine map_logarithm_glrlm_RunEntropy	0.589 (0.502, 0.676)	0.552 (0.414, 0.689)
	Venous phase iodine map_wavelet.LLH_gldm_GrayLevelVariance	0.523 (0.435, 0.610)	0.589 (0.457, 0.621)
	Venous phase mix image_original_firstorder_Skewness	0.604 (0.517, 0.690)	0.553 (0.414, 0.691)
Rad-score (VOI$^{outer\ layer}$)	Unenhanced iodine map_original_shape_MajorAxisLength	0.658 (0.570, 0.745)	0.589 (0.456, 0.723)
	Unenhanced mix image_wavelet.HHL_firstorder_Entropy	0.679 (0.593, 0.766)	0.672 (0.538, 0.805)
	Unenhanced mix image_wavelet.LLH_glcm_Idn	0.650 (0.562, 0.738)	0.645 (0.509, 0.781)
	Arterial phase iodine map_original_firstorder_Kurtosis	0.738 (0.683, 0.813)	0.690 (0.553, 0.827)
	Arterial phase iodine map_logarithm_glcm_DifferenceAverage	0.680 (0.592, 0.767)	0.615 (0.481, 0.749)
	Arterial phase iodine map_wavelet.HHH_gldm_LowGrayLevelEmphasis	0.610 (0.523, 0.710)	0.597 (0.465, 0.728)
	Arterial phase iodine map_wavelet.LHL_glcm_JointEnergy	0.666 (0.579, 0.754)	0.669 (0.583, 0.761)
	Arterial phase iodine map_wavelet.LLH_ngtdm_Complexity	0.623 (0.535, 0.710)	0.602 (0.515, 0.703)
	Arterial phase iodine map_square_glrlm_GrayLevelNonUniformity	0.664 (0.576, 0.752)	0.665 (0.550, 0.772)
	Arterial phase mix image_wavelet.HLH_firstorder_TotalEnergy	0.611 (0.524, 0.697)	0.608 (0.513, 0.688)
	Arterial phase mix image_wavelet.LHH_glrlm_GrayLevelVariance	0.643 (0.555, 0.731)	0.623 (0.531, 0.709)
	Venous phase iodine map_original_glszm_ZonePercentage	0.616 (0.528, 0.704)	0.621 (0.534, 0.717)
	Venous phase iodine map_wavelet.HLH_glcm_Idm	0.631 (0.544, 0.719)	0.611 (0.524, 0.701)
	Venous phase iodine map_wavelet.HLL_glrlm_HighGrayLevelRunEmphasis	0.600 (0.514, 0.686)	0.596 (0.564, 0.679)
	Venous phase mix image_exponential_firstorder_TotalEnergy	0.626 (0.539, 0.713)	0.614 (0.525, 0.708)
	Venous phase mix image_wavelet.LLL_glcm_Idm	0.581 (0.494, 0.669)	0.566 (0.441, 0.606)
Rad-score (VOI$^{inner\ layer}$)	Unenhanced iodine map_wavelet.LLH_glcm_DifferenceAverage	0.685 (0.598, 0.732)	0.625 (0.555, 0.666)
	Unenhanced mix image_original_shape_Flatness	0.665 (0.578, 0.753)	0.663 (0.558, 0.736)
	Arterial phase iodine map_original_gldm_DependenceEntropy	0.741 (0.667, 0.816)	0.736 (0.652, 0.808)
	Arterial phase iodine map_logarithm_glcm_SumAverage	0.706 (0.619, 0.793)	0.708 (0.623, 0.797)
	Arterial phase iodine map_logarithm_glszm_ZoneEntropy	0.675 (0.588, 0.762)	0.629 (0.557, 0.708)
	Arterial phase iodine map_wavelet.HLH_firstorder_Uniformity	0.621 (0.534, 0.708)	0.632 (0.574, 0.736)
	Arterial phase iodine map_wavelet.LHL_glcm_JointEnergy	0.697 (0.611, 0.784)	0.661 (0.602, 0.771)
	Arterial phase iodine map_log.sigma.3.0.mm.3D_gldm_LowGrayLevelEmphasis	0.689 (0.602, 0.776)	0.679 (0.600, 0.715)
	Arterial phase mix image_original_glrlm_GrayLevelNonUniformity	0.626 (0.538, 0.713)	0.633 (0.587, 0.759)
	Arterial phase mix image_squareroot_gldm_LowGrayLevelEmphasis	0.682 (0.595, 0.769)	0.669 (0.604, 0.779)
	Arterial phase mix image_wavelet.HLH_gldm_LowGrayLevelEmphasis	0.611 (0.524, 0.697)	0.606 (0.503, 0.673)
	Venous phase iodine map_original_glcm_Id	0.606 (0.519, 0.693)	0.612 (0.527, 0.697)
	Venous phase iodine map_square_firstorder_Energy	0.661 (0.573, 0.749)	0.658 (0.543, 0.727)
	Venous phase iodine map_logarithm_glszm_GrayLevelVariance	0.698 (0.611, 0.784)	0.688 (0.606, 0.734)
	Venous phase iodine map_wavelet.HLH_glrlm_HighGrayLevelRunEmphasis	0.682 (0.595, 0.769)	0.661 (0.542, 0.759)
	Venous phase mix image_original_glcm_Imc1	0.697 (0.610, 0.785)	0.664 (0.603, 0.754)
	Venous phase mix image_wavelet.HLL_firstorder_Kurtosis	0.610 (0.523, 0.698)	0.609 (0.517, 0.685)

Numbers in the parentheses are the 95% confidence interval. AUC, area under curve; CI, confidence interval; VOI, volume of interest.

Table S7. Performances of three different types of rad-scores for predicting initial recurrence risk in patients with PTC.

	AUC	Sensitivity	Specificity	PPV	NPV
**Training set**	-	-	-	-	-
**Rad-score (VOI^{whole})**	0.735 (0.661, 0.801)	0.810 (0.706, 0.890)	0.593 (0.482, 0.698)	0.646 (0.544, 0.740)	0.773 (0.653, 0.867)
**Rad-score (VOI$^{outer\ layer}$)**	0.794 (0.724, 0.853)	0.696 (0.582, 0.795)	0.779 (0.677, 0.861)	0.743 (0.628, 0.838)	0.736 (0.633, 0.823)
**Rad-score (VOI$^{inner\ layerr}$)**	0.837 (0.772, 0.890)	0.810 (0.706, 0.890)	0.767 (0.664, 0.852)	0.762 (0.657, 0.848)	0.815 (0.713, 0.892)
**Validation set**	-	-	-	-	-
**Rad-score (VOI^{whole})**	0.696 (0.576, 0.800)	0.690 (0.492, 0.847)	0.619 (0.450, 0.764)	0.643 (0.467, 0.775)	0.656 (0.481, 0.821)
**Rad-score (VOI$^{outer\ layer}$)**	0.827 (0.719, 0.906)	0.793 (0.603, 0.920)	0.833 (0.686, 0.930)	0.767 (0.577, 0.901)	0.854 (0.708, 0.944)
**Rad-score (VOI$^{inner\ layer}$)**	0.843 (0.737, 0.919)	0.793 (0.603, 0.920)	0.905 (0.774, 0.973)	0.852 (0.663, 0.958)	0.864 (0.726, 0.948)

Numbers in the parentheses are the 95% confidence interval. PTC, papillary thyroid cancer; AUC, area under curve; PPV, positive predictive value; NPV, negative predictive value; VOI, volume of interest.

Table S8. Stratification analysis of the final radiomics nomogram for predicting initial recurrence risk in PTC patients in different subgroups.

	AUC	Sensitivity	Specificity	PPV	NPV
**Age (y)**	-	-	-	-	-
**≤ 55**	0.873 (0.808, 0.922)	0.861 (0.759, 0.931)	0.733 (0.619, 0.829)	0.756 (0.649, 0.844)	0.846 (0.735, 0.924)
**> 55**	0.909 (0.679, 0.992)	0.857 (0.421, 0.996)	0.818 (0.482, 0.977)	0.750 (0.349, 0.968)	0.900 (0.555, 0.997)
**Age (y)**	-	-	-	-	-
**≤ 45**	0.862 (0.788, 0.918)	0.842 (0.721, 0.925)	0.739 (0.615, 0.840)	0.738 (0.615, 0.840)	0.842 (0.721, 0.925)
**> 45**	0.920 (0.796, 0.981)	0.864 (0.651, 0.971)	0.810 (0.581, 0.946)	0.826 (0.612, 0.950)	0.850 (0.621, 0.968)
**Gender**	-	-	-	-	-
**Male**	0.908 (0.795, 0.970)	0.964 (0.817, 0.999)	0.708 (0.489, 0.874)	0.794 (0.621, 0.913)	0.944 (0.727, 0.999)
**Female**	0.857 (0.778, 0.916)	0.706 (0.562, 0.825)	0.855 (0.742, 0.931)	0.800 (0.654, 0.904)	0.779 (0.662, 0.871)
**BMI (kg/m^2)**	-	-	-	-	-
**<25**	0.870 (0.795, 0.925)	0.683 (0.550, 0.797)	0.912 (0.807, 0.971)	0.891 (0.764, 0.964)	0.732 (0.614, 0.831)
**≥25**	0.909 (0.791, 0.973)	0.842 (0.604, 0.966)	0.931 (0.772, 0.992)	0.889 (0.653, 0.986)	0.900 (0.735, 0.979)

Numbers in the parentheses are the 95% confidence interval. PTC, papillary thyroid cancer; BMI, body mass index; AUC, area under curve; PPV, positive predictive value; NPV, negative predictive value.

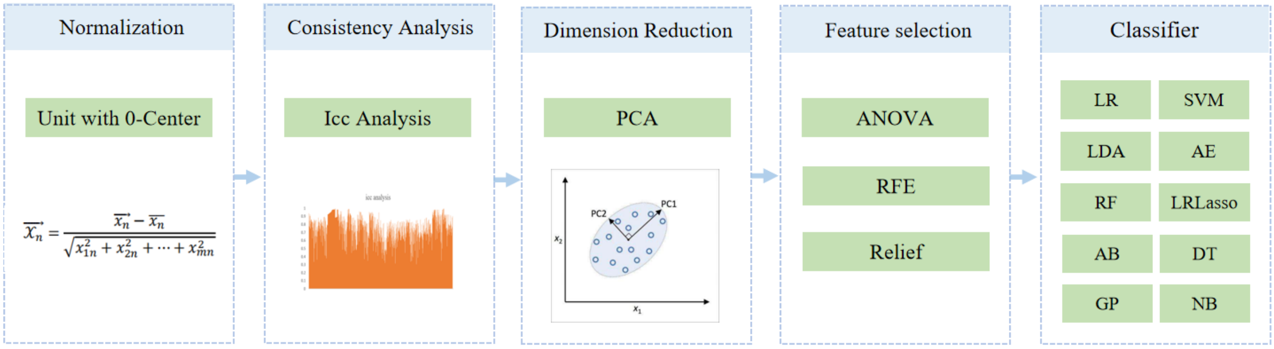

Fig. (S1). Our machine learning pipeline included data normalization, consistency analysis, dimension reduction, feature selection, and classifier modeling.

Notes: ICC, intraclass correlation coefficient; PCA, principal component analysis; ANOVA, analysis of variance; RFE, recursive feature elimination; LR, logistic regression; SVM, support vector machine; LDA, linear discriminant analysis; AE, auto-encoder; RF, random forest; LRLASSO, logistic regression via lasso; AB, ada-boost; DT, decision tree; GP, gaussian process; NB, native bayes.

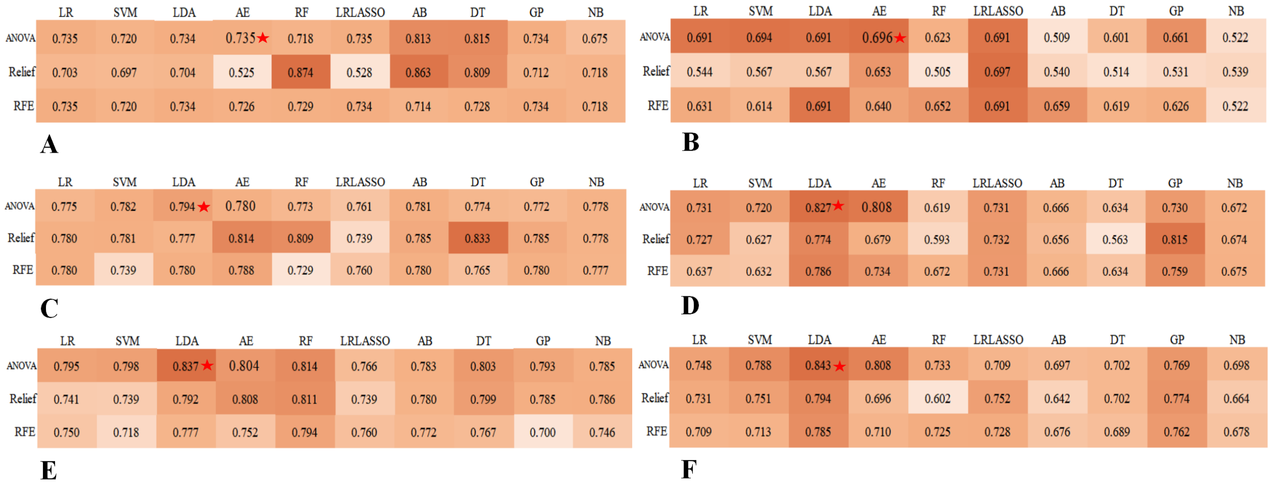

Fig. (S2). Heatmap of different combinations of radiomics features selection and machine learning classifier methods. The rows and columns displayed the different feature selection and machine learning classifier, respectively. The text embedded in the color code referred to AUC values for VOI^{whole} (**A**, **B**), VOI$^{outer\ layer}$ (**C**, **D**), and VOI$^{inner\ layer}$ (**E**, **F**) in the training and validation set, respectively. The optimal combination of feature selection method and classifier possessed the best predictive performance in both the training and validation set was marked with an asterisk.

Notes: ANOVA, analysis of variance; RFE, recursive feature elimination; LR, logistic regression; SVM, support vector machine; LDA, linear discriminant analysis; AE, auto-encoder; RF, random forest; LRLASSO, logistic regression via lasso; AB, ada-boost; DT, decision tree; GP, gaussian process; NB, native bayes.

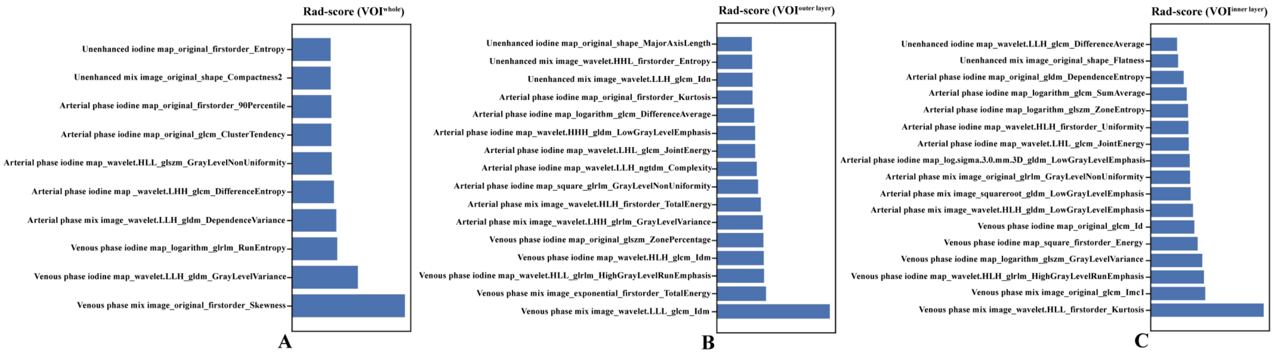

Fig. (S3). Bar chart illustrating the features and their corresponding weights in the rad-scores for different regions. (**A**) shows the top 10 radiomics features for rad-score (VOI^{whole}), (**B**) displays the top 16 radiomics features for rad-score (VOI$^{outer\ layer}$), and (**C**) presents the top 17 radiomics features for rad-score (VOI$^{inner\ layer}$). The bar height represents the feature's weight in the respective rad-score. VOI, volume of interest.

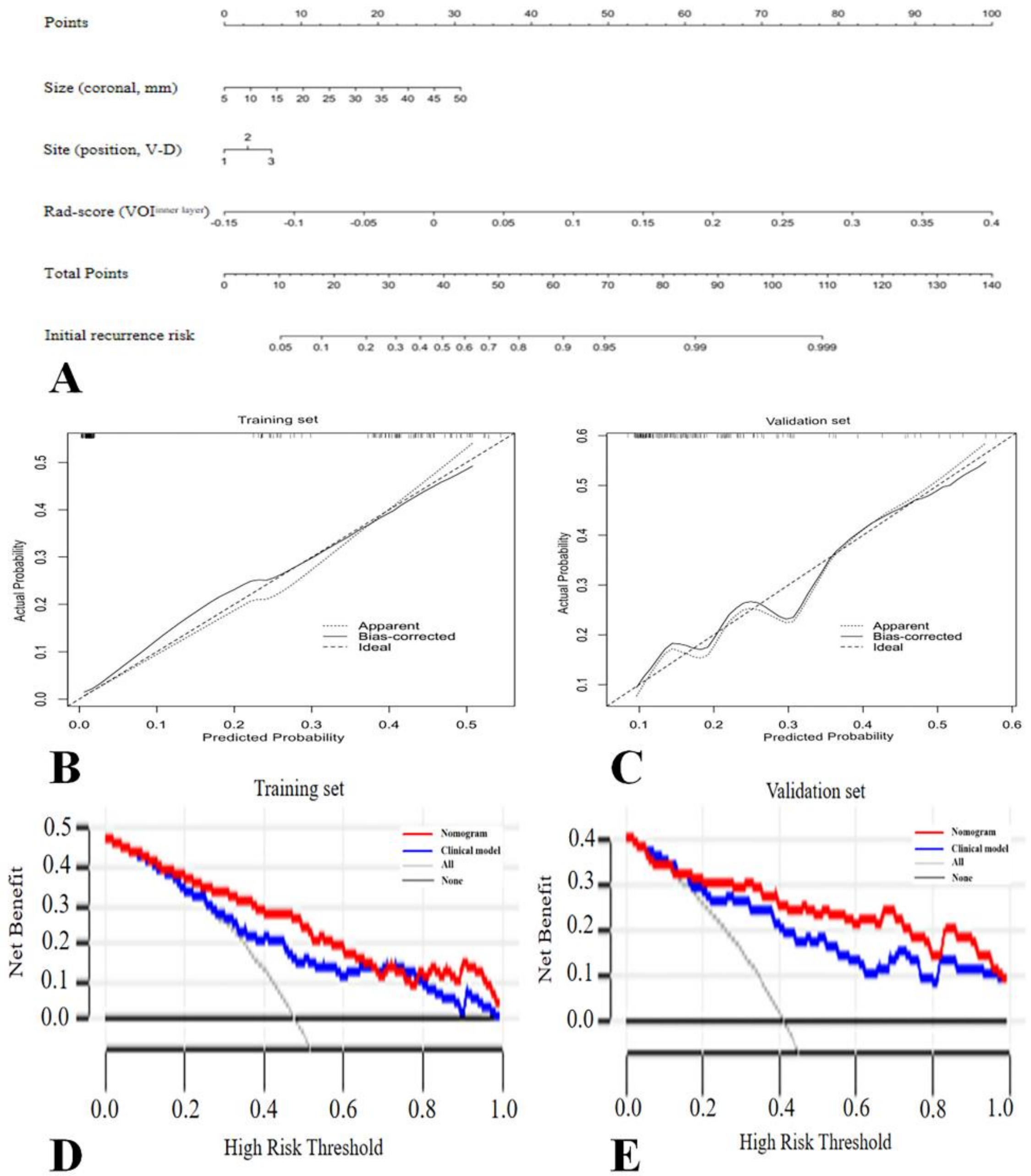

Fig. (S4). The radiomics nomogram construction and evaluation. The radiomics nomogram incorporated tumor size (coronal, mm), tumor site (position, V-D), and rad-score (VOI$^{inner\ layer}$) was built for clinical use (**A**). Calibration curves validated the nomogram good calibration in both the training (**B**) and validation (**C**) set. Decision curve analysis indicated that if threshold probability is between 0.165 and 0.682, the nomogram (red line) achieved more benefit than clinical model (blue line) in both the training (**D**) and validation (**E**) set. VOI, volume of interest.

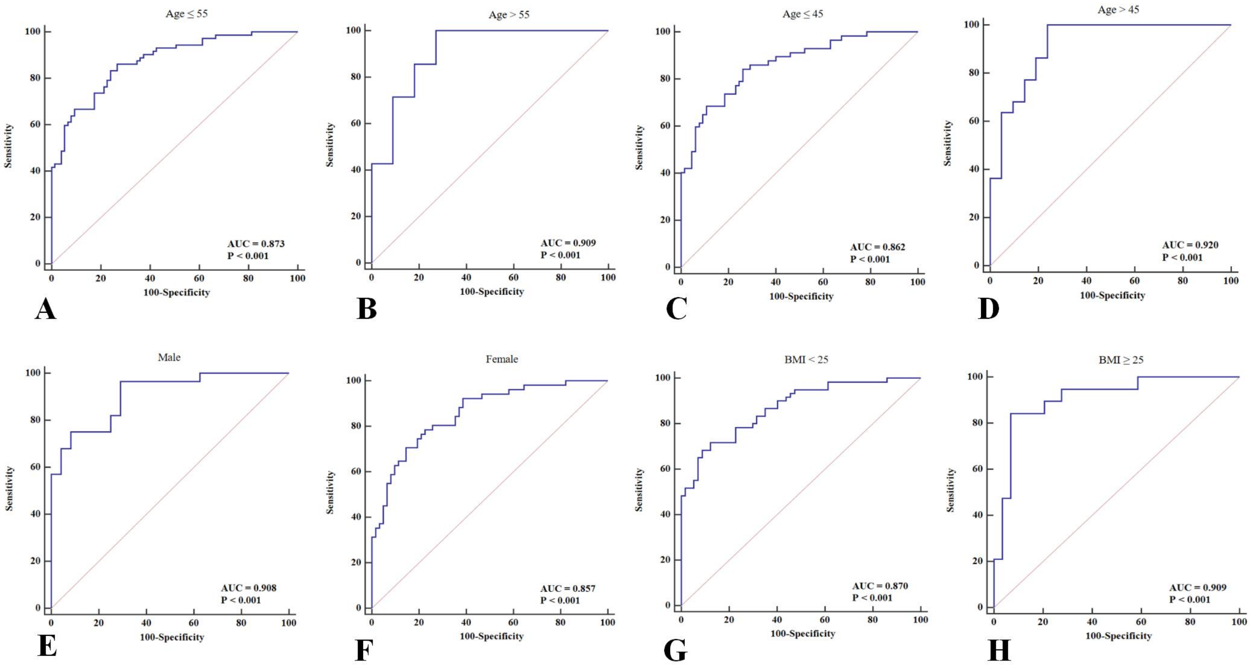

Fig. (S5). Predictive performances of the constructed nomogram in different sub groups according to age categorized by 55 (**A**, **B**), age categorized by 45 (**C**, **D**), gender (**E**, **F**), and BMI (**G**, **H**). BMI, body mass index.

© 2025 The Author(s). Published by Bentham Science Publisher.

This is an open access article distributed under the terms of the Creative Commons Attribution 4.0 International Public License (CC-BY 4.0), a copy of which is available at: https://creativecommons.org/licenses/by/4.0/legalcode. This license permits unrestricted use, distribution, and reproduction in any medium, provided the original author and source are credited.
